# Weight-Bearing Magnetic Resonance Imaging as a Diagnostic Tool That Generates Biomechanical Changes in Spine Anatomy

**DOI:** 10.7759/cureus.12070

**Published:** 2020-12-14

**Authors:** Brian Fiani, Daniel W Griepp, Jason Lee, Cyrus Davati, Christina M Moawad, Athanasios Kondilis

**Affiliations:** 1 Neurosurgery, Desert Regional Medical Center, Palm Springs, USA; 2 Neurosurgery, College of Osteopathic Medicine, New York Institute of Technology, Old Westbury, USA; 3 Medicine, College of Osteopathic Medicine, New York Institute of Technology, Old Westbury, USA; 4 Neurosurgery, Carle Illinois College of Medicine, University of Illinois at Urbana-Champaign, Champaign, USA; 5 Medicine, College of Osteopathic Medicine, Michigan State University, East Lansing, USA

**Keywords:** upright mri, weight bearing mri, spondylosis, clinical efficacy, cost effectiveness, neuroradiology, spinal stenosis, axial loading, dynamic mri, spine imaging

## Abstract

Weight-bearing magnetic resonance imaging (MRI) is a unique modality in diagnostic imaging that allows for the assessment of spinal pathology in ways considered previously inaccessible or insufficient with the conventional MRI technique. Due to limitations in positioning within the MRI machine itself, difficulties would be posed in determining the underlying cause of a patient’s pain or neurological symptoms, as the traditional supine position utilized can, in many cases, alleviate the severity of presented symptoms. Weight-bearing MRI addresses this concern by allowing a clinician to position a patient (to a certain degree) into flexion, extension, rotation, or side-bending with an axial load that can mimic physiologic conditions in order to replicate the conditions the patient experiences in order to give clinicians a clearer understanding of the anatomical relationship of the spine and surrounding tissues that may lead to a particular presentation of symptoms. These findings can then guide treatment approaches that are better tailored to a patient’s needs in order to initiate treatment earlier and shorten the duration of treatment necessary for patient benefit. The goal of this review is to describe and differentiate weight-bearing MRI from conventional MRI as well as examine the advantages and disadvantages of either imaging modality. This will include assessing cost-effectiveness and improvements in clinical outcomes. Further, the advancements of weight-bearing MRI will be discussed, including potentially unique clinical applications in development.

## Introduction and background

Magnetic resonance imaging (MRI) is an incredibly effective diagnostic tool for assessing spinal pathology [1]. Typically, this imaging modality captures images while the patient lays in a supine position without axial loading pressure on the spine. Although the supine position can increase comfort for the patient during extensive diagnostic scanning, difficulties are posed in that the positioning may not be sufficient to identify the relationship of the anatomic structures that are exacerbating a patient’s symptoms [2-4].

The technique of weight-bearing MRI was first described in 1997 by Willén et al. as a means to assess the morphological changes of tissue surrounding the spinal canal, particularly the dural sac and nerve roots [5]. Weight-bearing MRI provides a level of image detailing not typically present in traditional magnetic resonance imaging, in that it allows for the visualization of spinal morphology under axial load [5-7]. Visualization under these conditions is important when considering a patient’s chief complaint of back pain because it may only be incited in an upright or loaded position due to compression of the spinal canal or nerve roots, intervertebral instability, or disk degeneration, which may not be as prevalent under a decreased supine load [4,6]. Further, the dynamic nature of the weight-bearing MRI modality allows for positional adjustments of the patient to assess how rotation, flexion, extension, or side-bending can exacerbate the severity of symptoms for the patient in a dynamic fashion [6,8]. Herein, we review the mechanisms underlying conventional MRI and weight-bearing MRI as well as the advantages and disadvantages posed by either imaging modality. We provide additional insight into the long-term cost-effectiveness of diagnostic measures. Finally, future directions of these modalities will be explored, in particular, advances of the weight-bearing MRI technology and how it can continue to improve on clinical outcomes.

## Review

Technology and mechanism of action

**Figure 1:**
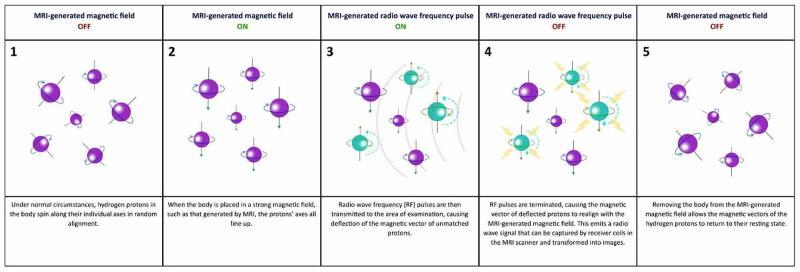
Summary of the molecular mechanism of action of MRI.

MRIs take advantage of the abundance of hydrogen ions in water and fat to generate an image based on their reaction to radio wave frequency (RF) pulses and magnetic fields. Powerful magnets are used to generate a strong magnetic field within the scanner. When a body is placed into the scanner, the nucleus of hydrogen atoms (protons) are forced into alignment with that field. This creates a magnetic vector oriented along the axis of the MRI scanner. An RF coil then emits RF pulses directed towards the target tissue, disrupting the alignment of the protons and placing them in a high-energy state. RF pulses are subsequently terminated, causing the realignment of the protons with the magnetic field (low-energy state) and the release of radio wave signals. The signal is captured by receiver coils in the scanner and transformed into images by the Fourier transformation algorithm [9-10]. The variation in the rate of relaxation between tissues allows them to be distinguished from one another in great detail. By specifying the rate of relaxation to be either the time taken for the magnetic vector to return to its resting state (T1) or the time needed for the axial spin to return to its resting state (T2), even greater emphasis can be placed on particular tissue or abnormalities [9]. A detailed summary of this process is depicted in Figure 1.

MRI scans have traditionally been performed with the patient in a recumbent position. However, weight-bearing MRI (WBMRI) is a relatively novel scanning technique that has the potential to replicate the dynamic components of lumbar spine degeneration, such as gravitational changes in lumbar degenerative morphology and segmental intervertebral instability. It has been shown to exaggerate particular degenerative disk morphologies (disc height, protrusion-herniation, and spinal stenosis) and, in some cases, identify pathologies not seen on conventional supine imaging [11-13]. This can be explained, in part, by the laws of fluid dynamics, which states that fluids are not compressible. Under physiologic axial loading, the disc space volume is reduced at levels of degeneration relative to the recumbent position. Compression of the semifluid discs leads to protrusion from the narrowed disc space and possible impingement of surrounding structures [11]. Furthermore, the significant increase in the lordosis lumbar angle within the upright position allows for the detection of position-dependent symptoms of spinal canal stenosis [12-13].

Advantages

The advantages of WBMRI include: 1) gauging mobility issues that are otherwise not distinguishable on conventional MRI, 2) accounting for gravitational forces that reproduce axial loading on the spine and joints, and 3) patient comfort in reducing feelings of claustrophobia [14].

First, a major advantage of WBMRI is the flexibility it provides in tracking various movements. This is incredibly useful in pain management, and specifically for patients suffering from lower back pain, segmental instability, and chronic conditions like sciatica [15]. The imaging systems’ multidirectional functionality provides visibility into incongruities generally undetected in a supine position. Maneuvers such as cervical spine flexion and extension are possible in weight-bearing positions in open MRI that potentially can offer additional information on cervical spine pathology and soft tissue width [15]. Substantial changes in bone orientation, intervertebral discs, ligaments, and tissue can be tracked in varying spinal positions. Many transforaminal imaging findings, such as intervertebral disc herniations and exit foraminal stenosis, can be dissected on weight-bearing MR examinations that aren’t visible on supine non-weight bearing examination [15]. This may benefit patients who have nerve root compression, as WBMRI may be a more sensitive test in highlighting variations in nerve root to disc abnormalities [5,16-17]. In comparison to supine imaging, deviations in nerve root contact can be visualized in weight-bearing positions [16,18]. Due to highly symptomatic neural compromise in patients suffering low-back pain, WBMRI provides additional measures in diagnosing nerve root compromise depending on their clinical findings [6,17].

Another advantage in the WBMRI is the accuracy of gauging the body in weight-bearing states, accounting for gravity that would exacerbate realistic vertebral compression in daily patient life. It allows either real-time kinetic or post-observational positional maneuvers of the examined body part in partial or full weight-bearing positions [15]. This is especially valuable when examining joint and spine abnormalities, as positional pain is produced in locations where weight is enforced [6]. This permits scanning accuracy in identifying positions where symptoms arise. This allows for a clearer diagnostic read than traditional, supine MRI, allowing for re-creating body positions that normally induce pain in a patient.

The WBMRI system has the potential to alleviate feelings of claustrophobia in comparison to the supine conventional MRI. WBMRIs allow the patient to stand or sit between imaging devices, with an open view of the room and minimal space enclosure. In a recent study by Eshed et al., MRIs requiring patients to lie supine while entering an enclosed scanner caused claustrophobia in 2% of patients undergoing imaging in comparison to a lower incidence in an upright weight-bearing position [14].

Disadvantages

Weight-bearing MRI has been shown to generate biomechanical changes in spine anatomy, which contribute to clinical correlations in spinal pathologies like spondylolisthesis, spinal stenosis, disc herniation, and more [7,19-21]. However, disadvantages have also been highlighted in a variety of studies, which are important to note when being used as a diagnostic tool for these pathologies. The use of weight-bearing MRI in the upright, seated, and simulated weight-bearing positions holds similar and unique disadvantages and challenges.

The upright standing MRI is known to have decreased magnetic strength as compared to traditional MRIs, which can correlate to lower image quality. Upright MRIs have a maximum field strength of 0.6 Tesla [T] Fonar Upright MRI (Fonar Corporation, Melville, New York), which is the major determinant of image quality [22]. Comparable MRIs used for conventional imaging in the supine position are commonly 3T [23]. This reduction in field strength results in a reduced signal to noise ratio [7,24]. Lower strength MRI can potentially lead to longer scanning times, which can increase motion artifacts [15]. Imaging the patient in the positioning of symptomatology may lead to increased motion leading to poor and decreased image quality [25]. In addition, difficulty can sometimes be encountered in evaluating the most lateral areas of the spine such as the foramen and lateral recesses [24].

The ability of the upright MRI to obtain images of the spine under stress allows for the diagnosis of pathologic conditions that only manifest under stress [7,11,24]; however, this does not aid in positioning for surgical interventions This disadvantage requires the need for re-imaging with a conventional supine MRI prior to surgery in patients who are diagnosed via upright MRI, which can drive up cost and resource allocations [22]. Currently, MRI in the supine position is widely available in medical facilities around the world, but upright MRI is only available at select locations, which poses a disadvantage of accessibility to these machines [22-23]. As more studies identify possible diagnostic use, weight-bearing MRI is still not widely available. This can lead to challenges in the future to practice the standard of care for institutions that do not have these devices yet. In addition, there is no specific billing code for upright/positional MRI, which can lead to multiple “views” being billed separately, driving up the cost [22]. In addition, insurance companies may be hesitant to reimburse a second MRI [8]. The proposed benefits of the upright MRI has the potential to further complicate cost, quality, and efficiency when and if it is transitioned into wide-scale clinical use.

Weight-bearing MRI has also been shown to demonstrate biomechanical changes not visualized in the standard supine position, which can sometimes pose a disadvantage. In 2008, Madsen et al. showed a significant decrease in lumbar lordosis during standing MRI when compared to lying, which can be due to precautions taken to secure and immobilize for the vertical position [20]. The weight-bearing MRI in the upright seated position has been shown to underestimate the true extent of disease due to relative flexion of the lumbosacral junction, as compared with the standing position [7]. Of note, the upright-seated position is noted to be in partial flexion [22]. In addition, the supine weighted-simulated position with the DynaWell harness (Dynawell Diagnostics, Inc., Henderson, Nevada) has been shown to induce increased curvature of the spine via Cobb angle measurements as compared to the upright position [26]. Similarly, spinal stenosis that can be uncovered in the erect position may not always correlate to clinical symptoms [27]. This can lead to unwarranted surgical intervention with delayed treatments [28]. Lastly, in the case of the cervical spine, Vitaz et al. in 2004 showed minor additional benefits of upright MRI and do not recommend it for replacing conventional supine MRI for non-complex cases [8].

Trials, outcomes, and cost

In order to better identify the use of WBMRI, a literature search of national databases was performed to identify studies that reported changes to the spine during loading. Studies were also identified that could provide insight into the clinical use and specific indications of WBMRI. Although studies including WBMRI (true weight-bearing, upright, or seated) were preferred, studies reporting on supine MRI with axial-load induced by a DynaWell L-spine compression harness were also included. Twenty-two studies published over the last 20 years on 6,871 patients were identified as having meaningful data that looked at the clinical utility as well as dynamic and static changes of the spine during loading. Pathologies included spinal stenosis, spondylolisthesis, disc herniation, scoliosis, and neurogenic claudication, as well as generalized back pain and degenerative changes. Twelve studies reported data on WBMRI (true, seated, or standing), and 10 studies reported on supine, “simulated” axial-loaded MRI using a DynaWell harness. The studies identified are listed and summarized in Table 1.

**Table 1:** Outcomes of weight-bearing magnetic resonance imaging (MRI) as a modality in diagnostic imaging T, Tesla; DCSA, dural cross-sectional area; BW, Bodyweight; L, Lumbar; C, Cervical; DS, Degenerative spondylolisthesis; SpS, Spinal stenosis

Author, Year; Journal	N	Patient Pathology	MRI Setup	Position, Weight-Bearing	Outcome Summary
Danielson, 2001 [29]; Spine	43	Asymptomatic	1.0-T, (Magnetom Impact; Siemens, Munchen, Germany) with Dynawell Harness compression device	Supine, simulated 50% BW	Reduction to 76% of unloaded DCSA
Kimura, 2001 [30]; Spine	8	Asymptomatic	1.5-T system (Magnetom Vision; Siemens, Erlangen, Germany) with Dynawell Harness compression device	Supine, simulated 50% BW	Kyphotic angle change at L3-L4 and L5-S1; Disc height change only at L4-L5
Willen, 2001 [31]; Spine	172	Low back pain; Sciatica; Neurogenic claudication	1.0-T, (Magnetom Impact; Siemens, Munchen, Germany) with Dynawell Harness compression device	Supine, simulated 50% BW (and) Upright, true	50/172 showed a significant reduction of the DCSA, increased disc herniation, lateral recess, or foraminal stenosis
Vitaz, 2004 [8]; Southern Medical Journal	20	Cervical neck pain	0.5-T Signa SP/i interventional MRI system (GE Medical Systems, Milwaukee, WI)	Seated, true	Weight-bearing MRI in extension specifically showed significant cord compression in C-spine
Wessberg, 2006 [26]; Spine	30	Scoliosis	1.5-T MR scanner (Gyroscan Intera, Eindhoven, The Netherlands) with Dynawell Harness compression device	Supine, simulated 50% BW (and) Upright, true	Significant increase of Cobb angle with upright versus simulated loading
Karadimas, 2006 [2]; Journal of spinal disorders & techniques	30	Degenerative Lumbar spine	0.6-T FONAR Upright MRI (Melville, New York, NY)	Seated, True	Statistically significant reduction of endplate angle and disc height
Hirasawa, 2007 [32]; Spine	29	Asymptomatic	0.6-T FONAR Upright MRI (Melville, New York, NY)	Upright, true	23.8% reduction of DCSA
Perez, 2007 [33]; European Journal of Radiology	89	Posterior disc herniation; Spondylolisthesis;	0.6-T FONAR Upright MRI (Melville, New York, NY)	Seated, true	Missed pathology identified in 52/89 case of supine vs. seated MRI
Madsen, 2008 [20]; Spine	36	Lumbar spinal stenosis	0.2-T (Siemens Open Viva, Siemens, Erlangen, Germany	Supine, simulated 50% BW	Extension with axial loading showed a significant reduction of DCSA
Hansson, 2009 [18]; European Spine journal	24	Lumbar Lumbo-sacral pain	1.5-T system using a surface coil with Dynawell Harness compression device	Supine, simulated 50% BW	Ligamentum flavum bulging caused 50-85% of spinal canal narrowing
Gilbert, 2011 [21]; Journal of Manipulative and Physiological Therapeutics	1486	Foraminal, lateral recess, or central stenosis	Airis II (Hitachi Medical Systems, Twinsburg, OH) low-field (0.3 T) for recumbent MRI and midfield (0.6 T) for Upright MRI	Upright, true	Detection rate of Stenosis shown to be 38.5% (recumbent) and 56.7% (weight-bearing)
Niggemann, 2012 [34]; Skeletal Radiology	50	Juxtafacet cysts (JFC)	0.6-T FONAR Upright MRI (Melville, New York, NY)	Upright, true	The detection rate of JFC improves with increased lordosis of the L spine
Ozawa, 2012 [35]; Journal of Neuroradiology	88	Degenerative spondylolisthesis; Spinal stenosis;	1.5-T system (Magnetom Vision; Siemens, Erlangen, Germany) with Dynawell Harness compression device	Supine, simulated 50% BW	Axial loading showed a significant change in DCSA in patients with degenerative spondylolisthesis compared with spinal stenosis
Tarantino, 2013 [24]; J Orthopaedic Traumatology	57	Low back pain	0.25-T tilting system (G-scan Esaote)	Upright, true	Upright MRI showed hidden disc protrusions and/or spondylolisthesis in 70% of cases
Kim, 2013 [36]; Spine	54	Spinal Canal stenosis	1.5-T MR scanner (Gyroscan Intera, Philips Systems, Best, The Netherlands) with Dynawell Harness compression device	Supine, simulated 50% BW	13/54 patients showed a reduction of DCSA
Segebarth, 2015 [37]; Journal of spinal disorders & techniques	109	Degenerative spondylolisthesis	N/A	Upright, true	Nearly 1/3 of spondylolisthesis missed on supine MRI
Nguyen, 2016 [38]; Journal of craniovertebral junction & spine	17	Asymptomatic; Lumbar back pain; Radiculopathy;	0.6-T FONAR Upright MRI (Melville, New York, NY)	Seated, true	Axial loading of MRI showed the most significant change when the patient was symptomatic
Splendiani, 2016 [13]; La Radiologia Medica	4305	Back pain	0.25-T tilting system (G-scan Esaote)	Upright, true	hidden modifications of protrusions and/or herniated discs detected in 66% of upright imaging
Lau, 2017 [27]; European Spine journal	70	Neurogenic claudication	0.25-T low-field MR system (G-scan, Esaote, Genoa, Italy) weight-bearing platform with a hydraulic tilting mechanism	Upright, true	DCSA significantly reduced and correlated with higher clinical pain scores
Kanno, 2018 [39]; The Spine Journal	41	Degenerative Spondylolisthesis	1.5-T system (Magnetom Vision; Siemens, Munich, Germany) with Dynawell Harness compression device	Supine, simulated 50% BW	DCSA significantly reduced with a strong correlation with the degree of listhesis
Lang, 2018 [40]; Cureus	10	Lumbar degeneration	0.25-T open-configuration scanner with rotatable examination bed allowing for true standing MRI	Upright, true	Neural foraminal and central canal volume reduced; sagittal listhesis and lumbar lordosis increased
Sasani, 2019 [41]; World Neurosurgery	103	Neurogenic claudication;	1.5-T MR scanner (Gyroscan Intera, Philips Systems, The Netherlands) with Dynawell Harness compression device	Supine, simulated 50% BW	DCSA significantly reduced

Study outcomes

Most studies that identified changes in the axial-loaded spine reported a reduction in dural cross-sectional area (DCSA) as the primary outcome of interest [20,24,27,29,32,35,39,41-42]. This was radiographically most apparent in most studies and perhaps an expected change of a loaded spine with known degenerative changes, including spinal stenosis. It was expected that pathologies most related to a significant reduction of DCSA would include degenerative spondylolisthesis and spinal canal stenosis, which are known to cause pressure on the spinal cord and induce neurogenic claudication [30,41]. However, the majority of studies showed a reduction of DCSA regardless of significant medical history, known degenerative or pathological spinal pathology, and presence or absence of symptoms.

Disc height and endplate angle changes in supine versus WBMRI were also studied [2,30]. Of the two studies reporting on this, one reported simulated axial load with the DynaWell harness and one reported the use of WBMRI (true, seated). While the study with simulated axial loading showed only disc height reduction in L4-L5 [32], the WBMRI study showed both reductions in disc height at multiple levels as well as a reduction of endplate angles in the lumbar spine [2]. This indicated that true WBMRI was potentially more efficacious in producing results that would be more clinically useful when compared with the DynaWell harness. Three studies also measured the degree of listhesis in supine versus WBMRI and showed statistically significant increased listhesis in patients with preexisting degenerative change or spondylolisthesis [24,40,43]. Another study of scoliosis patients showed increased Cobb angle in WBMRI [26].

Because the literature reported mostly on changes in the lumbar spine, only a few studies were identified that considered weight-bearing changes in the thoracic or cervical spine. Understandably, these levels do not have as great a significant change during WBMRI due to the lower weight of the body structures they support. However, one WBMRI study, in particular, did notice a significant narrowing of the cervical spinal canal, particularly in extension, accompanied by a reproduction of cervical pain during the course of imaging [8].

Clinical significance of outcome measures

DCSA, which is effectively a transverse measure of the spinal canal opening, is a complex outcome measure, as reduction occurs to a certain degree just from physiological changes in a loaded spine. Furthermore, it may occur, perhaps to a greater degree, in patients with a single spinal pathology or multiple co-existing spinal pathologies. In studies of patients with pre-existing pathology, the structural causes of DCSA reduction were mostly attributed to the extension of the lumbar spine in the setting of spinal stenosis [44]. The dynamic causes of spinal canal narrowing seemed more related to soft tissue changes in the canal. Hansson et al. concluded of patients with lumbosacral pain that the major cause of spinal canal narrowing was due to the ligamentum flavum and not disc protrusion [18]. Splendiani et al. showed that in patients with pre-existing lumbar pain, hidden disc protrusions and spinal modifications were the primary cause of pain [13]. Thus, while DCSA was shown to be reduced in each study of both asymptomatic and symptomatic patients, there is little consensus on the most likely contributors to that change. While WBMRI can potentially uncover an underlying cause of pain or pathology, an individualized approach to each patient would be required to compare supine MRI to WBMRI in order to correlate the clinical picture with DCSA reduction.

As mentioned previously, the degree of lumbar lordosis is another important consideration. Reduction in DCSA or degree of spinal stenosis may also be attributed to a greater degree of lumbar lordosis, which is a known physiological change of a loaded spine [44]. In light of this concept, Hansen et al. studied the use of a lumbar pillow to induce a similar physiological change that would occur during WBMRI and assessed whether or not observed changes were comparable in both approaches (WBMRI and pillow) [45]. Findings were suggestive of induced lumbar lordosis with a pillow not being suitable to induce the same degree of spinal stenosis, shown by using a semi-quantitative grading scale. While the decrease in DCSA was comparable, the degree of spinal stenosis was greater in the WBMRI group, indicating that WBMRI was a more sensitive test for spinal stenosis compared to other measures (such as a lumbar pillow) that simply reduce DCSA or induce lumbar lordosis. This further strengthened the use of WBMRI, rather than other modalities such as a lumbar pillow or external compressive devices, to induce true weight-bearing load on the spine in producing the most accurate clinical picture. This study was significant because it confirmed that changes in the spine seen in WBMRI were not solely due to the increase in the degree of lumbar lordosis but also due to the increase in weight.

In further analysis of spinal stenosis, one retrospective study completed by Gilbert et al. examined the change in the detection rate of stenosis between WBMRI and supine MRI [21]. While WBMRI imaging showed a significantly increased detection rate in the subcategories of lateral recess, foraminal, and central stenosis, this study had limitations. Although all patients identified were symptomatic, given this study was retrospective, it was difficult to understand the indications that led to a patient receiving WBMRI. It is possible that a more severe clinical picture guided the provider to pursue WBMRI studies, which would then undoubtedly show higher detection rates of stenosis in a subgroup of patients who were more symptomatic [21]. Stenosis detection rate as a direct measure of DCSA was also studied using simulated axial loading with the DynaWell harness. Madsen et al. and Ozawa et al. described this in a well-controlled clinical setting where a supine MRI could be completed, followed by immediate re-scanning with an axial load applied on the DynaWell harness. Despite previously described limitations of the failure of the harness to induce true physiologic loading and increased lumbar lordosis, DCSA was still significantly reduced [20-21]. Notably, each of these changes was observed in the presence of pre-existing spinal pathology and, as such, these findings do not necessarily show that WBMRI is useful in identifying spinal stenosis in every patient. On the contrary, it would seem to indicate the specific utility of WBMRI in showing the higher sensitivity and specificity of spinal stenosis rates only in patients with known pre-existing spinal degeneration or pathology.

In the discussion of spinal change in asymptomatic individuals, three studies with meaningful results were identified [29,30,32]. Outcomes of interest were DCSA, reduction in disc height, and degree of stenosis, with changes observed due to the physiologically expected response to loading of the spine. It was interesting that these studies of asymptomatic individuals showed a reduction of DCSA, often to a comparable degree of changes observed in symptomatic patients [29,30,32]. This seemed to indicate a potential lack of clear clinical correlation with DCSA and spinal pathology. Lau et al. completed a study that seemed to challenge this idea by showing a strong correlation between patient pain scores and reduction in DCSA [27]. However, this study was completed in patients with known neurogenic claudication. Clinical pain scores would undoubtedly be higher in the standing position during the WBMRI, which is when DCSA would also be reduced. Thus, the correlation was expected simply due to the nature of the underlying pathology. If a similar study was repeated in asymptomatic patients, the study could undoubtedly conclude that there is no correlation between DCSA and patient pain scores.

Together, these studies suggest that physiological postural changes could be the most likely contributors to decreased DCSA, and detection by WBMRI does not offer meaningful clinical findings in the absence of symptoms. Thus, a clinician employing the use of WBMRI should have a high suspicion that changes observed in the spine during WBMRI, such as decreased DCSA, may or may not be the source of a patients’ symptoms and does not always indicate degenerative changes. Based on these findings, the authors of this review believe WBMRI should be used primarily in conjunction with supine MRI only in symptomatic patients with known spinal pathology to rule in or rule out specific differential diagnoses. This supports the findings of previous studies that have shown WBMRI does allow for the linkage of a symptomatic patient’s clinical syndrome with possible spinal pathology, which suggests improved sensitivity and specificity of the WBMRI [11].

In-hospital considerations and cost-effectiveness

If a neurosurgical department sought to regularly assess patients through some form of MRI observing loading-bearing changes of the spine, the most cost-effective way would be with the use of a DynaWell harness with a pre-existing supine MRI device. While the downsides of this approach have previously been addressed, such as failure to induce physiological lumbar lordosis or the ability of the harness to apply only 50% of a patients’ body weight [34], it does allow for a relatively cost-effective way to elicit obvious changes in a loaded spine. Notably, in out-patient settings, the option is available to have imaging done at a dedicated imaging facility, which may have a 0.6 Tesla [T] Fonar Upright MRI device or comparable WBMRI. However, during in-hospital settings, where the patient is already admitted to the service, a physician would be limited to the use of hospital imaging. If routine supine and WBMRI imaging were desired within hospital departments, this would necessitate having two dedicated MRI devices.

Recent online searches and contact with MRI distributers revealed the similar cost of upright versus supine MRI, with both sharing a similar spectrum of minimum and maximum cost. Some studies reported estimates of MRI scanners to have a nominal cost of $1 M per tesla (T) of the magnetic field [46]. In general, the fixed costs related to purchasing and establishing an MRI was estimated to be between 1-2 million dollars; the administrative cost was estimated on average to be almost one-hundred thousand dollars [47]. Some of this cost is due to the necessary addition of a dedicated MRI room, which is another significant cost to construct within a hospital. As discussed previously, true WBMRI is most useful for patients with known spinal degeneration, thus, these steps would only be a worthy investment if the patient caseload was high in that particular hospital.

Furthermore, although a hospital or provider may deem a WBMRI necessary, it is considered a separate imaging test that would have to be justifiable to insurance companies, as discussed in the disadvantages section. Blackmore et al. reported that insurance companies had found insufficient evidence of the cost-effectiveness of WBMRI, citing a lack of clinical research showing clear clinical utility in 2009 [48]. The DynaWell harness was also discussed as an alternative, given that it may induce compressive changes in the spine and did not require the use of an entirely different machine [48]. Some additional concerns cited by Chung et al. (mentioned in the disadvantages section) included: 1) possible reduced quality due to the lower field strength of upright MRI machines (despite this strength being described as optimal for this purpose) and 2) potential for MRI devices to generate multiple images in a single exam, which may be billed separately, unnecessarily increasing costs [22]. Chung et al. estimated that, on average, the increased number of scans being billed per patient visit at upright MRI facilities in Washington State was 2.5 times higher than conventional, supine MRI facilities, concluding that diversification of MRI devices, in general, was potentially further magnifying the known problem of high cost in medical imaging [22].

## Conclusions

Weight-bearing MRI allows for a range of testing possibilities that would not be possible with conventional MRI. Given the ability to dynamically position a patient into extension, flexion, rotation, or side-bending under axial load, a clinician is able to replicate the physiologic conditions that incite a patient’s symptoms and allows for the evaluation of the unique anatomical relationship between the spine and adjacent structures that underlies a patient’s specific clinical presentation. The collection of studies in this review promotes the notion of WBMRI as an effective tool in assessing an array of spinal pathologies with a higher degree of specificity and sensitivity as compared to other imaging modalities.

As the technology continues to advance, WBMRI will need to be tested for its ability to be incorporated into artificial intelligence models for predicting ideal spine surgery candidates. Such models are being utilized with conventional MRI imaging, but randomized prospective trials are needed to determine if image-capturing under high-stress loading holds true and contributes to patient selection for surgery. Future dynamic radiographic testing holds promise in guiding the diagnosis and treatment of joint pathologies such that surgical interventions can be better tailored to address the weight distribution and compression of the affected joint the patient experiences under physiologic conditions in order to improve long-term clinical outcomes. The effective application of WBMRI under current diagnostic criteria, along with potential future advancement into additional anatomical regions, supports the continued use of this unique imaging modality in patient care.
